# The relationship between self-enhancing humor and precuneus volume in young healthy individuals with high and low cognitive empathy

**DOI:** 10.1038/s41598-018-21890-0

**Published:** 2018-02-22

**Authors:** Bingbing Li, Xu Li, Yangu Pan, Jiang Qiu, Dajun Zhang

**Affiliations:** 1 School of Psychology, Southwest University, Chongqing, 400715 China; 2 Center for Mental Health Education, Southwest University, Chongqing, 400715 China; 3 Department of Psychology, Shanghai Normal University, Shanghai, 200234 China; 4 Research Institute of Social Development, Southwest University of Finance and Economics, Chengdu, 610074 China; 5 Key Laboratory of Cognition and Personality (SWU), Ministry of Education, Chongqing, 400715 China

## Abstract

A self-enhancing humor style (SEHS) plays an important role in the regulation of negative emotion through humorous perspective-taking. Following the mind-reading theories of humor, we investigated the relationship between gray-matter volume (GMV) of brain areas related to theory of mind and SEHS in young college students, using voxel-based morphometry analysis. We then performed a voxel-wise analysis of covariance to assess any moderation effect of cognitive empathy on the relationship. Results demonstrated that higher SEHS scores were associated with larger GMV of the precuneus in the group with high cognitive empathy, but there was no association in the group with low cognitive empathy. These results suggest that high cognitive empathy and increased precuneus volume can predict greater use of self-enhancing humor in young healthy individuals.

## Introduction

Humor, that certain psychological state that tends to produce laughter, plays an essential role in social settings. “Sense of humor” refers to humor as a stable personality trait^1^. A self-enhancing humor style (SEHS), which may be measured via a subscale of the Humor Styles Questionnaire (HSQ)^2^, involves a generally humorous outlook on life, and maintaining a humorous perspective even in the face of stress or adversity^3^. SEHS is negatively related to anxiety, depression, and bad mood; it can be regarded as a coping mechanism or adaptive defense^4^.

Several functional magnetic resonance imaging (fMRI) studies have examined the brain regions involved in humor^5^, and have revealed the brain regions that are most consistently activated across individuals. However, in personality research, the main focus has been on inter-individual differences. Magnetic resonance imaging (MRI) studies utilizing voxel-based morphometry (VBM) analysis can reveal regional variation in brain volume related to individual differences in psychological traits^6^. Therefore, in present study we examined whether regional variation in gray matter volume (GMV) was associated with SEHS in a large sample of college students.

## Association between SEHS and GMV of brain areas related to theory of mind

Mind-reading theories of humor claim explicitly that theory of mind (ToM) abilities are necessary to process humor^7,8^; such theories are supported by both behavioral and neuroimaging studies. At the behavioral level, regression analyses indicate that poor-mind reading (one subscale of the autism-spectrum quotient) is associated with lower SEHS scores in the general population^9^, and individuals with Asperger’s syndrome, who have limited ToM ability, use less self-enhancing humor than control groups^10^. A recent fMRI study demonstrated that the processing of self-enhancing humor primarily activates the brain regions involved in ToM, such as the medial prefrontal cortex, precuneus (PCU), and the temporo-parietal junction^11^. Therefore, we hypothesized that SEHS scores would be associated with the GMV in ToM related brain areas (Hypothesis 1).

## The moderation effect of cognitive empathy on the neural basis of SEHS

ToM describes the ability to represent other people’s mental states, such as beliefs, desires, emotions, and goals in order to predict their actions^12^. Empathy is a complex form of psychological inference in which observation, memory, knowledge, and reasoning are combined to yield insights into the thoughts and feelings of others^13^. Empathy involves both the ability to share the emotional experience of the other person (i.e. the affective component) and an understanding of the other person’s experience (i.e. the cognitive component)^14^. In other words, the emphasis of affective empathy is typically placed on experiencing the emotional states of others, whereas the cognitive empathy focuses more on attributing the feelings of others. Therefore, cognitive empathy is very closely related to ToM^15^, and is sometimes described as perspective taking or ToM^16^.

Converging evidence suggests that the relationship between the GMV of ToM-related areas and SEHS may be moderated by different levels of cognitive empathy. First, the conceptions of ToM and cognitive empathy overlap to a certain extent. Previous research has demonstrated that patients with schizophrenia and individuals with autism, who are characterized by ToM deficits, also experience difficulty with cognitive empathy^17–20^. Furthermore, brain areas involved in the processing of cognitive empathy and perspective taking, such as the medial prefrontal cortex, PCU, and temporo-parietal junction, are also activated in ToM-based tasks^15,21,22^. Second, cognitive empathy can influence humor appreciation that requires the juxtaposition of mental states (ToM). Specifically, empathizers provide more mentalistic explanations as to why they think a cartoon is funny than do systemizers^23^. Individuals with autism and schizophrenia exhibit impaired humor appreciation^24,25^, especially of ToM cartoons^26–29^. Taken together, we speculate that there will be a positive association between the GMV of ToM-related areas and SEHS in individuals with high cognitive-empathy (i.e., above the average score), whereas the association will disappear in individuals with low cognitive-empathy (i.e., below the average score; Hypothesis 2).

In the present study, cognitive empathy was measured by the dimension “appraisal of the emotion of other” (AEO) in the Chinese version of the Emotional Intelligence Scale^30^. Empathy is one key component of trait Emotional Intelligence^31–33^, and the AEO subscale of the Emotional Intelligence Scale contains items that might be regarded as measuring cognitive empathy. Examples of such items include “I know what other people are feeling just by looking at them” and “I am aware of the non-verbal messages other people send.”

## Results

### Sample characteristics

Two participants were excluded because of incomplete questionnaire data. Another five participants were omitted from further analyses due to extraordinary motion artifacts. There remained 279 participants whose data were analyzed further.

The demographic data and the distribution of psychometric measures in the high and low cognitive empathy groups are shown in Table 1.

**Table 1:** Descriptive statistics of behavioral results for high and low cognitive empathy groups.

	High CE group (n = 129, 67 males)		Low CE group (n = 150, 62 males)		Group difference	
	*Mean*	*SD*	*Mean*	*SD*	*T score*	*P*
Age	20.05	1.44	19.93	1.29	0.73	0.46
CRT	66.44	3.31	66.36	3.10	0.21	0.83
AEO	16.42	1.35	13.38	1.63	22.29	<0.00**
SEHS	24.36	6.00	22.56	5.93	2.51	0.01*

Note: CE, cognitive empathy; CRT, Combined Raven’s Test; AEO, appraisal of the emotion of other; SEHS, self-enhancing humor style; *SD*, standard deviation.

*P < 0.05; *P < 0.01.

### VBM results

**Figure 1:**
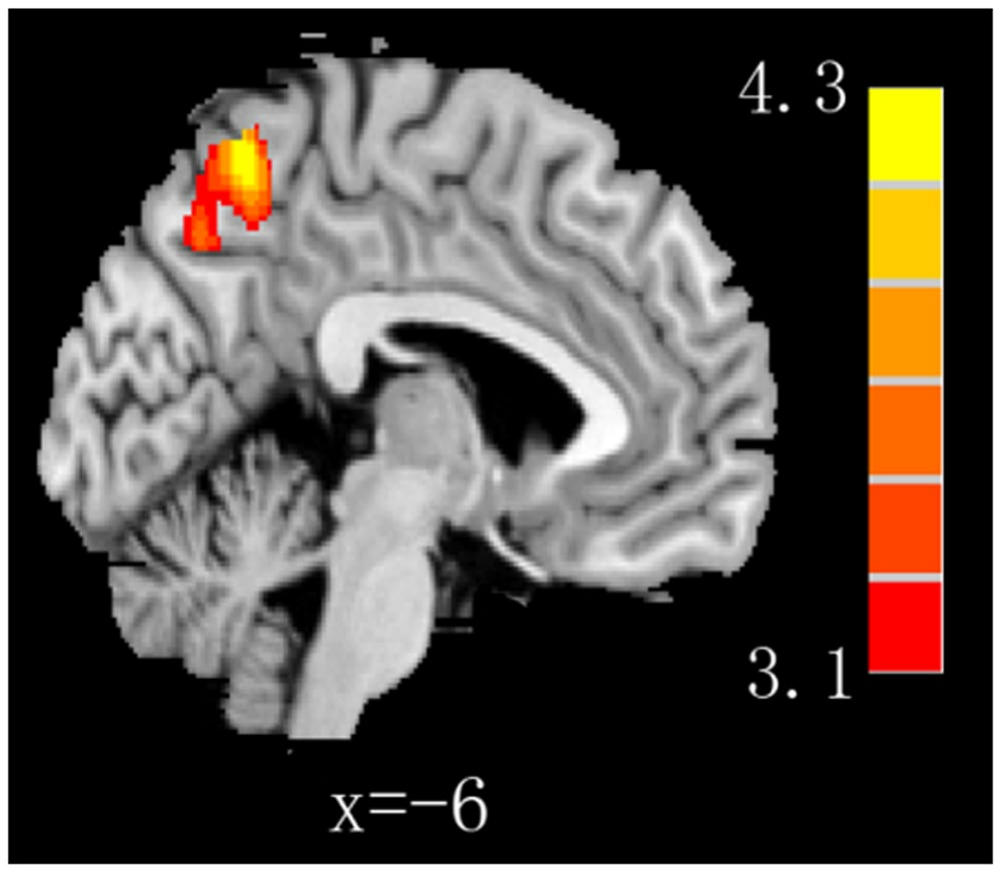
An interaction effect between cognitive empathy group and SEHS found in regions of the bilateral PCU. SEHS, self-enhancing humor style; PCU, precuneus.

There was no significant association between regional GMV and SEHS. Interestingly, an interaction effect between cognitive empathy and SEHS (t-contrasts of the effect of SEHS score for high and low cognitive empathy group were [1-1]) was found in regions of the bilateral PCU (x = −6, y = −59, z = 56; *t* = 4.23, *p* < 0.05; cluster size 1814 voxels; Fig. 1). These results indicate that the correlation between SEHS and the GMV of the PCU in the high cognitive empathy group was significantly greater than that in the low cognitive empathy group. No other significant effects were found.

To gain more insight into the moderated effect, we conducted a simple moderation model (Model 1) using the PROCESS procedure of SPSS^34^. First, the GMV of the PCU of each participant was extracted by the Resting-State fMRI Data Analysis Toolkit software package^35^. Second, the GMV of the PCU, and the raw scores of AEO and SEHS were converted to z scores to reduce any multicollinearity. Third, in the analysis model, the GMVs of the PCU were entered as independent variables, the scores of SEHS as dependent variables, the AEO scores as the moderator variables, while age, gender, total GMV, and general intelligence were regarded as covariates.

**Figure 2:**
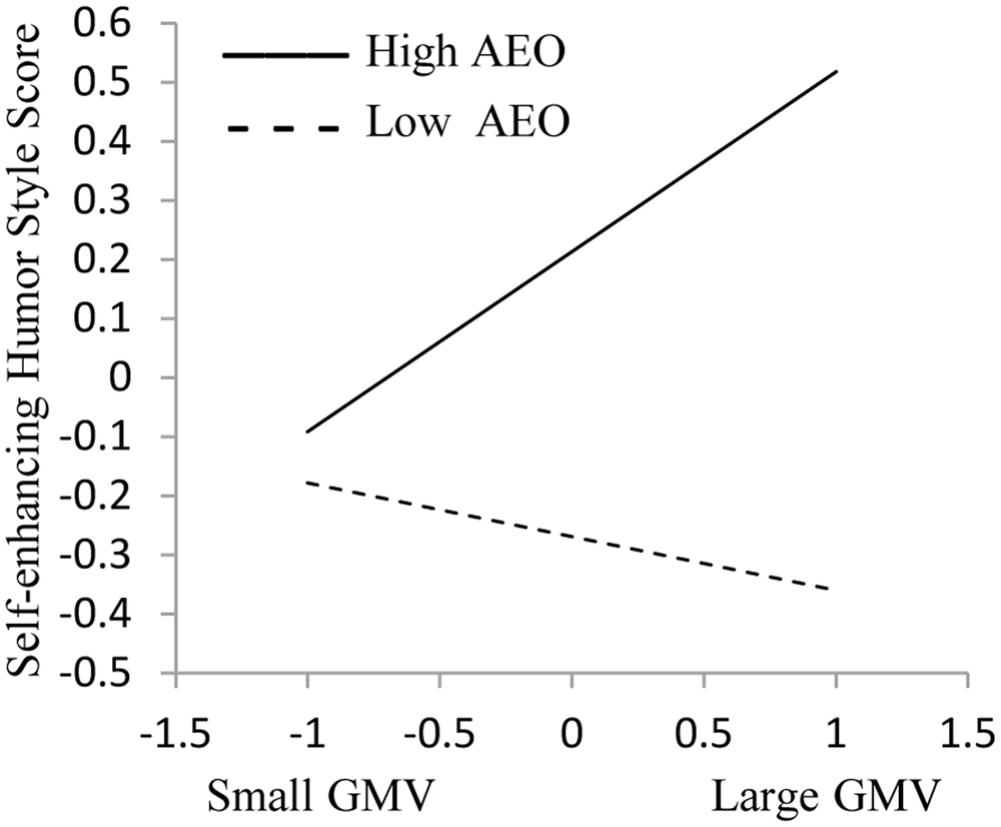
The moderation effect of AEO on the relationship between SEHS and the GMV of PCU. The x-axis represents standardized scores of the GMV of PCU and the y-axis represents standardized SEHS scores. The solid line indicates the high AEO group and the dashed line indicates the low AEO group. AEO, appraisal of the emotion of other; SEHS, self-enhancing humor style; GMV, gray matter volume; PCU, precuneus.

The moderation analysis demonstrated that there was a significant interaction between the GMV of the PCU and the AEO scores, *β* = 0.18, *SE* = 0.06, *t* = 2.88, *p* < 0.01. Simple slope analysis revealed that for participants with high AEO scores (1 SD above the mean), the effect of the GMV of the PCU on SEHS was significant, *β* = 0.23, *SE* = 0.11, 95% CI = [0.02, 0.44], *t* = 2.16, *p* < 0.05, whereas for participants with low AEO scores (1 SD below the mean), the effect of the GMV of the PCU on SEHS did not reach significance, *b* = −0.14, *SE* = 0.10, 95% CI = −0.34, 0.06], *t* = −1.36, *p* = 0.17 (Fig. 2).

## Discussion

In the present study, we investigated the relationships among the GMV of brain areas related to ToM and SEHS in a large sample. We further explored the moderating role of cognitive empathy on the relationships. Our results demonstrated that only in the high cognitive empathy group was the GMV of PCU significantly associated with SEHS; no association was found in the low cognitive empathy group. We statistically controlled for age, gender, total GMV, and general intelligence, each of which could have plausibly exerted a confounding influence on our research.

Consistent with the mind-reading theories and our prediction, SEHS showed an association with the GMV of ToM-related brain regions, and cognitive empathy moderated this relationship. Specifically, there was a significant association between the GMV of the PCU and SEHS in the high cognitive empathy group, whereas the association was missing in the low cognitive empathy group. Previous neuroimaging studies demonstrated that the PCU is involved in the mental processes related to ToM, cognitive empathy, and perspective-taking^21,36–39^, which is essential to humor processing^7,8^, and perspective-taking has a closer relationship with SEHS than other humor styles measured by HSQ^40^. Several fMRI studies have demonstrated that activation of the PCU is associated with the processing of humor requiring mentalization^41,42^. For example, compared to neutral pictures, humorous cartoons increase activity in the PCU, angular gyrus, and thalamus^41^. Furthermore, a recent fMRI study reported that the PCU is activated while processing self-enhancing humor^11^. These associations are evidence of the importance of structural properties of the PCU for SEHS.

The function of the PCU may be restricted in individuals with low cognitive empathy, in whom there is no relationship between the GMV of the PCU and SEHS, while for individuals armed with high cognitive empathy, the function of PCU promotes their adoption of a humorous perspective on life, as demonstrated by the result that larger GMV of PCU was associated with higher SEHS scores. First, there was no difference in the GMV of PCU between high and low cognitive-empathy groups (*t* = 0.26, *p* = 0.79), but SEHS scores were greater in the high cognitive-empathy group (*t* = 2.51, *p* < 0.05). Second, the PCU is abnormally activated in patients with ASD or schizophrenia; both conditions are characterized by impaired cognitive-empathy abilities. For example, in resting-state fMRI research, patients with autism spectrum disorder show decreased functional-connectivity between the PCU and medial-prefrontal cortex relative to controls^43^. The PCU has been observed to exhibit hypoactivation in a perspective-taking task in patients with schizophrenia^44^. Taken together, this evidence suggests that high cognitive empathy may be beneficial to develop the function of the PCU, which contributes to the individual’s increased use of self-enhancing humor.

Of note, we found no relationship between other ToM-related brain areas and SEHS, and cognitive empathy did not moderate the relationship between the medial prefrontal cortex and SEHS. This is surprising because the medial prefrontal cortex, posterior superior temporal sulcus, and anterior temporal region play an important role in mentalization^45^, which is implicated in humor processing related to ToM. For example, Gallagher, *et al*.^46^ found that the medial prefrontal cortex is activated in ToM compared to non-ToM cartoon and story tasks. Samson, *et al*.^47^ reported that ToM cartoons, compared to semantic cartoons, increased activation in the mentalizing areas, such as medial prefrontal cortex, posterior superior temporal sulcus, and PCU. Our results may be consistent with the observation that individual differences in anatomical structures and blood-oxygenation-level dependent activity may be dissociated for components of brain networks associated with certain behaviors^6^.

Some limitations of this study should be acknowledged. The moderation effect of cognitive empathy should be further investigated in future studies. For example, one might investigate the performance of individuals with different levels of cognitive empathy, using a cartoon-joke fMRI paradigm. In addition, given that we recruited young healthy subjects, it is not clear whether the current findings would generalize to the full range of population variation, such as individuals with a decline in ToM abilities associated with normal aging^48^.

In conclusion, the present study examined associations between brain areas related to ToM and SEHS using VBM methods in a large sample, and the moderation effect of cognitive empathy on the association. Our results revealed a significant positive association between the GMV of the PCU and SEHS in the high cognitive empathy group, whereas there was no association in the group with low cognitive empathy. These findings suggest that high cognitive empathy together with increased PCU volume can predict higher SEHS scores.

## Methods Subjects

In total, 286 healthy right-handed undergraduate or postgraduate students were recruited from the local community of Southwest University (China). None of the subjects reported a prior history of neurological or psychiatric disease, or substance abuse. This study was approved by the Institutional Review Board of the Southwest University Imaging Center for Brain Research. The experiment procedure accorded with the standards of the Declaration of Helsinki. All participants provided written informed consent prior to taking part in the study. A battery of psychological instruments were completed by volunteers, including the HSQ, Emotional Intelligence Scale, and Combined Raven’s Test^49^.

### Assessment of self-enhancing humor style

We measured SEHS using the self-enhancing humor style subscale (5 items) of the HSQ^2^. In the Chinese version of the HSQ, Cronbach’s alpha for the SEHS was reported as 0.78^50^. In the present study, Cronbach’s alpha, was 0.78.

### Assessment of cognitive empathy

Cognitive empathy was measured by the AEO subscale of the Emotional Intelligence Scale. The revised Emotional Intelligence Scale has good psychometric qualities and can be applied to Chinese samples^30^. The AEO subscale of the Emotional Intelligence Scale (5 items) was used to measure the ability to appraise the emotions of others through verbal or non-verbal information (e.g., “I am aware of the non-verbal messages other people send”). In the present study, the Cronbach’s alpha coefficient was 0.74.

### Assessment of general intelligence

To account for the effect of general intelligence on brain structures^51^, the Combined Raven’s Test^52^ was used to assess each individual’s intelligence. The Combined Raven’s Test, which has a high degree of reliability and validity^49^, consists of 72 items as revised by the Psychology Department of East China Normal University in 1988. The total score of this test (number of correct answers in 40 min) was used as a psychometric index of individual intelligence^53^.

### Imaging data acquisition

Structural magnetic resonance imaging (MRI) data were acquired using a 3.0-T Siemens Trio MRI scanner (Siemens Medical, Erlangen, Germany). T1-weighted anatomical images were acquired using a magnetization-prepared rapid gradient-echo sequence (repetition time = 1900 ms, echo time = 2.52 ms, inversion time = 900 ms, flip angle = 9 degrees, 256 × 256 matrix, slices = 176, slice thickness = 1.0 mm, and voxel size = 1 × 1 × 1 mm^3^).

### VBM analysis

VBM was employed to characterize the differences in GMV to determine the neuroanatomical correlates of behavioral performance across participants^54^. The structural image data were processed using the SPM8 software (Wellcome Department of Cognitive Neurology, London, UK; www.fil.ion.ucl.ac.uk/spm) implemented in Matlab 7.8 (MathWorks Inc., Natick, MA, USA). First, structural images were displayed in SPM8 to check for artifacts or gross anatomical abnormalities. Second, the images were reoriented manually to the anterior commissure. Third, T1-weighted anatomical images were segmented into gray matter and white matter using a unified segmentation approach^55^. Fourth, we performed diffeomorphic anatomical registration through exponentiated Lie algebra in SPM8 for registration, normalization, and modulation^56^. Fifth, to preserve the volume of tissue in each structure, the image intensity of each voxel was modulated by the Jacobian determinants. Sixth, registered images were transformed to Montreal Neurological Institute space. Finally, the normalized and modulated images were smoothed with a 10-mm full-width at half-maximum Gaussian kernel to increase their signal to noise ratio.

### Statistical analysis

Statistical analysis of imaging data was performed using SPM8. In the analysis, we used whole-brain multiple-regression analysis to identify regions where regional GMV was associated with individual differences in SEHS. In the multiple regression analysis, the SEHS scores were used as the variable of interest, whereas gender, age, total GMV, and general intelligence were entered as the covariates of no interest.

We were also interested in whether the relationships between the GMV of brain areas related to ToM and SEHS differed between high and low cognitive empathy groups. Therefore, in the whole-brain analysis we used a voxel-wise analysis of covariance (ANCOVA), with cognitive empathy as a grouping factor (using the one-way ANOVA option of SPM8). High and low cognitive empathy groups were defined with reference to the mean score of the AEO subscale. This method has been successfully employed in previous studies^57–59^. In this analysis, age, gender, global GMV, general intelligence, and SEHS were covariates. All covariates, except global GMV, were modeled so that each covariate had a unique relationship with GMV for each group (using the interaction option in SPM8), which enabled investigation of the effects of the interaction between cognitive empathy and each covariate. The global GMV was not modeled in this manner, and a common effect of global GMV on regional GMV was assumed for both cognitive empathy groups. In these analyses, the centering option was used for centering the interaction. The interaction between cognitive empathy and SEHS (contrasts of [the effect of SEHS score for high and low cognitive empathy group] were [1-1] or [−1 1]) were assessed using t-contrasts.

For the VBM analysis and the ANCOVA analysis, an absolute voxel signal intensity threshold masking of 0.2 was used to minimize gray matter/white matter boundary effects. The significance threshold was set at a voxel-wise *p* < 0.001 uncorrected and a cluster-level threshold of *p* < 0.05 family-wise error corrected at the whole-brain level.

### Data availability

The datasets generated during the current study are available from the corresponding author on reasonable request.
